# DOCU-CLIM: A global documentary climate dataset for climate reconstructions

**DOI:** 10.1038/s41597-023-02303-y

**Published:** 2023-06-23

**Authors:** Angela-Maria Burgdorf, Stefan Brönnimann, George Adamson, Tatsuya Amano, Yasuyuki Aono, David Barriopedro, Teresa Bullón, Chantal Camenisch, Dario Camuffo, Valérie Daux, María del Rosario Prieto, Petr Dobrovolný, David Gallego, Ricardo García-Herrera, Joelle Gergis, Stefan Grab, Matthew J. Hannaford, Jari Holopainen, Clare Kelso, Zoltán Kern, Andrea Kiss, Elaine Kuan-Hui Lin, Neil J. Loader, Martin Možný, David Nash, Sharon E. Nicholson, Christian Pfister, Fernando S. Rodrigo, This Rutishauser, Sapna Sharma, Katalin Takács, Ernesto T. Vargas, Inmaculada Vega

**Affiliations:** 1 Institute of Geography, University of Bern, Bern, Switzerland; 2 Oeschger Centre for Climate Change Research, University of Bern, Bern, Switzerland; 3 Department of Geography, King’s College London, London, UK; 4 School of Biological Sciences, The University of Queensland, Brisbane, Australia; 5 Graduate School of Agriculture, Osaka Metropolitan University, Osaka, Japan; 6 Instituto de Geociencias (IGEO), CSIC-UCM, Madrid, Spain; 7 Department of Geography, Autonomous University of Madrid (UAM), Madrid, Spain; 8 Institute of History, Department of Economic, Social and Environmental History (WSU), University of Bern, Bern, Switzerland; 9 National Research Council-Institute of Atmospheric Sciences and Climate, Corso Stati Uniti 4, Padua, Italy; 10 Laboratoire des Sciences du Climat et de l’Environnement, CNRS, CEA, UVSQ, Université Paris-Saclay, Gif-sur-Yvette, France; 11 Argentine Institute of Nivology, Glaciology and Environmental Sciences (IANIGLA-CONICET), Mendoza, Argentina; 12 Facultad de Filosofía y Letras, Universidad Nacional de Cuyo, Mendoza, Argentina; 13 Department of Geography, Faculty of Science, Masaryk University, Brno, Czech Republic; 14 Global Change Research Institute, Czech Academy of Sciences, Brno, Czech Republic; 15 Departamento de Sistemas Físicos, Químicos y Naturales, Universidad Pablo de Olavide, Seville, Spain; 16 Departamento de Física de la Tierra y Astrofísica, Universidad Complutense, Madrid, Spain; 17 IGEO, Instituto de Geociencias (CSIC, UCM), Madrid, Spain; 18 Fenner School of Environment and Society, Australian National University, Canberra, Australia; 19 ARC Centre for Climate Extremes, The Australian National University, Canberra, Australia; 20 School of Geography, Archaeology and Environmental Studies, University of the Witwatersrand, Johannesburg, South Africa; 21 Department of Geography, College of Science, University of Lincoln, Lincoln, UK; 22 Natural Resources Institute Finland, Helsinki, Finland; 23 Department of Geography, Environmental Management and Energy Studies, University of Johannesburg, Johannesburg, South Africa; 24 Institute for Geological and Geochemical Research, Research Centre for Astronomy and Earth Sciences, Budapest, Hungary; 25 CSFK, MTA Centre of Excellence, Konkoly Thege Miklós út 15-17, Budapest, Hungary; 26 Institute of Hydraulic Engineering and Water Resources Management, Vienna University of Technology, Vienna, Austria; 27 Research Center for Environmental Changes, Academia Sinica, Taipei, Taiwan; 28 Graduate Institute of Environmental Education, National Taiwan Normal University, Taipei, Taiwan; 29 Department of Geography, Swansea University, Swansea, UK; 30 Department of Biometeorological Applications, Czech Hydrometeorological Institute, Prague, Czech Republic; 31 School of Applied Sciences, University of Brighton, Brighton, United Kingdom; 32 Department of Earth, Ocean, and Atmospheric Science, Florida State University, Tallahassee, Florida USA; 33 Department of Chemistry and Physics, University of Almería, Almería, Spain; 34 Department of Biology, York University, Toronto, Ontario Canada; 35 Institute for Soil Sciences (TAKI) and Centre for Agricultural Research (ATK), Budapest, Hungary; 36 National Museum of Natural Sciences-Spanish Research Council, Madrid, Spain

**Keywords:** Palaeoclimate, Atmospheric science

## Abstract

Documentary climate data describe evidence of past climate arising from predominantly written historical documents such as diaries, chronicles, newspapers, or logbooks. Over the past decades, historians and climatologists have generated numerous document-based time series of local and regional climates. However, a global dataset of documentary climate time series has never been compiled, and documentary data are rarely used in large-scale climate reconstructions. Here, we present the first global multi-variable collection of documentary climate records. The dataset DOCU-CLIM comprises 621 time series (both published and hitherto unpublished) providing information on historical variations in temperature, precipitation, and wind regime. The series are evaluated by formulating proxy forward models (i.e., predicting the documentary observations from climate fields) in an overlapping period. Results show strong correlations, particularly for the temperature-sensitive series. Correlations are somewhat lower for precipitation-sensitive series. Overall, we ascribe considerable potential to documentary records as climate data, especially in regions and seasons not well represented by early instrumental data and palaeoclimate proxies.

## Background & Summary

Information on past climates has played an essential role in climate science^1^. While historically the primary research focus has been on reconstructing past annual temperature, the questions raised nowadays with the help of palaeoclimatological data are multifaceted, including changes in the water cycle, the occurrence of weather and climate extreme events, and atmospheric dynamics. This, in turn, is a challenge for producing palaeoclimatic datasets. New approaches, such as off-line palaeodata assimilation^2–5^, provide past climate fields at increasing spatial and temporal resolution. However, all reconstructions essentially depend on sufficient high-quality data inputs.

Current climate field reconstructions are largely based on relatively high-resolution proxies measured in natural archives such as tree rings, corals, speleothems, bivalves, sediments, or ice cores. Extensive compilations of such proxies exist^6,7^. In particular, tree rings are widely used, among others, due to their extensive spatial distribution across the globe. Unlike most other natural proxies, tree ring proxies, have an annual resolution. However, their climate signal is mainly limited to the growing season (although there are also winter reconstruc tions^8^). Documentary proxies, i.e., climate data originating from historical documents, could provide an essential contribution since they potentially cover combinations of seasons and regions (e.g., winter in East Asia) that are otherwise not well represented by natural proxies. Furthermore, documentary data are often calendar dated, and some have a very high temporal resolution. Despite these advantages, they are largely overlooked and only marginally used in large-scale climate reconstructions, since they are not readily available in digital format in the main compilations used by climate scientists, or their quality is not well known (note that classification of events is often based on effects, which requires local context information). The PAGES 2k multiproxy database^9^, for instance, only includes 15 documentary proxy series. However, historians have compiled documentary climate information in databases such as EURO-CLIMHIST for many years^10^ (note that qualitative weather descriptions are also found in databases of early instrumental meteorological data, e.g., Rodrigo^11^).

In recent years, a major international effort has been done to promote the use of the archives of societies in climate reconstructions. The PAGES CRIAS working group (Climate Reconstruction and Impacts from the Archives of Societies) was founded in 2018 and is working towards that goal. The Palgrave Handbook of Climate History^12^ provided a first global overview of documentary climate data organized in regional chapters. Based on this and many other sources, Burgdorf^13^ recently inventoried documentary climate series from a literature and databases search. The inventory contains 688 entries; not all are publicly available, and some have not yet been digitized. Here, we publish a subset of the data inventoried in Burgdorf^13^, termed DOCU-CLIM. The dataset contributes to a global monthly palaeoclimate reanalysis starting in 1420 and is based on assimilating monthly-to-seasonal proxies, documentary data and instrumental data into an ensemble of atmospheric model simulations using an offline Kalman filter approach similar to Valler *et al*.^5^ For that reason, here we focus on series that provide information in the window 1400–1880 CE at monthly to annual resolution. In this paper we present the dataset (see Supplementary File DOCU-CLIM_Inventory.txt for an overview of all records) and evaluate its usefulness for climate reconstruction using proxy forward models (statistical models that predict the documentary series from climate data rather than vice versa).

## Methods

### Compilation and data rescue

Over the past decades, climate historians and historical climatologists have produced numerous datasets in which documentary data have been translated into quantitative climate information. However, as their focus is commonly regional or local, these data are often not submitted to global data repositories such as the NOAA World Data Service for Palaeoclimatology database (https://www.ncei.noaa.gov/access/paleo-search/) but instead, published on project or personal websites, or, unfortunately still very often, not published at all. Even if documentary records are incorporated into databases, they may not always be organized in a manner suitable for climate scientists, particularly when working with time series. In this work, we focus exclusively on quantitative document-based time series data, representing a small, albeit underexploited, subset of the body of documentary climate data.

**Fig. 1:**
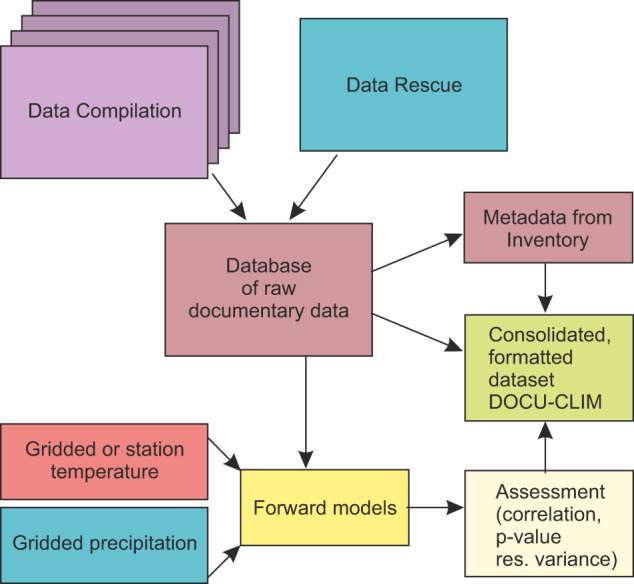
Flow chart depicting the generation of the documentary dataset.

Figure 1 illustrates the general workflow followed in this project. The compilation of an inventory of documentary climate series was described in a previous paper^13^, which lists 688 records. While the latter paper described the metadata, in this paper we compiled the actual data. As detailed in Burgdorf^13^, the inventory was based on a search of 14 existing databases (Table 1), contributing about 25% of the entries in the inventory, as well as extensive literature research, contributing the rest. The catalogued data followed a set of criteria, some of which were dictated by our intended use. For instance, we only inventoried material overlapping the period 1400–1880 CE, with a minimum record length of 30 years of which 20 must be before 1880. These criteria were set as we used the data in a data assimilation project starting in 1420 CE and in which instrumental data (which become more frequent after 1880 CE) were also assimilated. The variables of interest were temperature, precipitation, and wind (e.g., onset of seasonal wind regime) and hence only records were compiled that provide information on one of these variables (note that some of the series also depend on further variables). As detailed in Burgdorf^13^, the focus was predominantly on English-language literature that was accessible electronically and in which the authors state that the series contains information on one of the three variables (publications about the Mediterranean area and Central Europe in other languages exist and may contain additional series). Except for phenological data, we used only secondary material to ensure the inclusion of expert source interpretation. This includes derived indices, generally accepted to quantify descriptive and qualitative documentary data^14^ or even reconstructed time series in physical units.

**Table 1:** Overview of available global and national repositories and databases containing documentary evidence.

Name of repository or database	Abbreviation	Region	N_all_	N_docu_	Reference	URL
PAGES2k Global 2,000 Year Multiproxy Database	PAGES2k	Global	692	15	Emile-Geay *et al*.^6^	https://www.ncei.noaa.gov/ access/paleo-search/study/ 21171 (last access: 30 May 2022)
NOAA/World Data Service for Paleoclimatology archives	NOAA Paleo	Global	>10000	61		https://www.ncei.noaa.gov/ access/paleo-search/ (last access: 30 May 2022)
Euro-Climhist	Euro-Climhist	Switzerland/central Europe	65	27	Pfister *et al*.^10^	https://www.euroclimhist.unibe.ch/en/ (last access: 30 May 2022)
Tambora.org	Tambora.org	Germany	4	4	Riemann *et al*.^133^	http://www.tambora.org (last access: 30 May 2022)
National Snow and Ice Database: Global Lake and River Ice Phenology	NSIDC	Northern Hemisphere	865	39	Benson *et al*.^25^, updated 2020	https://nsidc.org/data/g01377/versions/1 (last access: 30 May 2022)
Japan Climate Data Project	JCDP	Japan	14	3		https://jcdp.jp (last access: 30 May 2022)
Climatological Database for the World’s Oceans	CLIWOC	Global	1624		García-Herrera *et al*.^134^	https://www.historicalclimatology.com/cliwoc.html (last access: 30 May 2022)
Institute for Ocean Technology Ice Database	Ice Data	Canada	4	4		http://www.icedata.ca (last access: 30 May 2022)
KNMI Climate Explorer	Climate Explorer	Global	>200	~10		https://climexp.knmi.nl/ start.cgi?id = someone@ somewhere (last access: 30 May 2022)
Red Española de Reconstrucción Climática a Partir de Fuentes Documentales	RECLIDO	Spain	7	7		http://stream-ucm.es/RECLIDO/es/home-es.htm (last access: 30 May 2022)
Salvá Sinobas	Salvá Sinobas	Iberian Peninsula	18	5		http://salva-sinobas.uvigo.es/index.php (last access: 30 May 2022)
Variabilidad y Reconstrucción del Clima	Vareclim	Global	5	5		https://www.upo.es/ vareclim/index.php (last access: 30 May 2022)
Reconstructed East Asian Climate Historical Encoded Series	REACHES	China	1	1	Wang *et al*.^26^	https://www.ncdc.noaa. gov/paleo- search/study/ 23410 (last access: 30 May 2022)
Tracking Extremes of Meteorological Phenomena Experienced in Space and Time	TEMPEST	United Kingdom	5	5	Veale *et al*.^135^	https://www.nottingham. ac.uk/research/groups/ weather-extremes/research/ tempest-database.aspx (last access: 30 May 2022)

N_all_ is the total number of series or databases available on the platform, and N_docu_ is the number of those series based on documentary evidence and available prior to 1880 CE (from Burgdorf^13^). Note that there may be overlap between the repositories.

The next step was to compile the actual data. Not all inventoried series are available in electronic form, and some are subject to a restrictive data policy. We downloaded the series from 14 databases (Table 1) and contacted many authors directly in cases when a dataset was not available in a repository. However, we only compiled data series that are open access and allow us to redistribute the data under a CC-BY license.

**Fig. 2:**
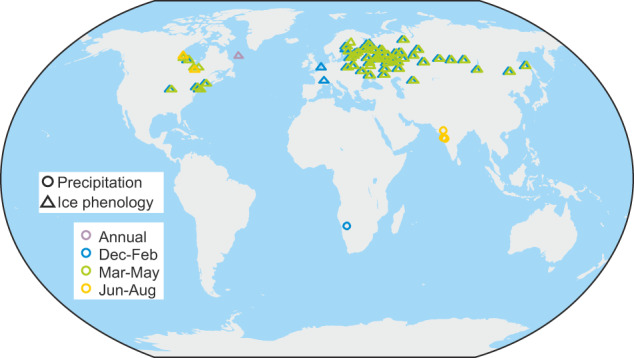
Map of rescued series.

**Fig. 3:**
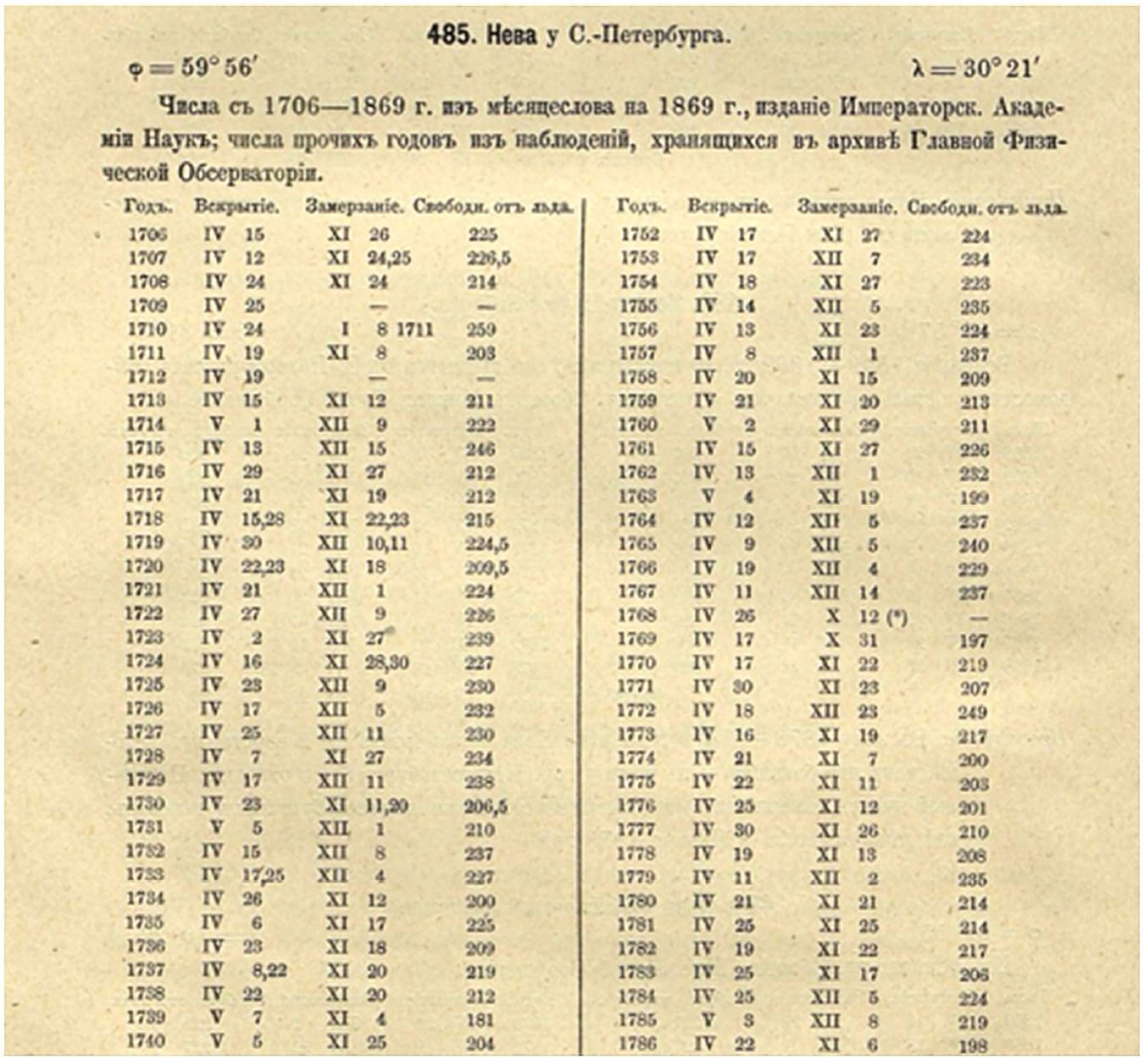
Extract from Rykachev^16^ showing the dates of freezing and thawing of the Neva River in St. Petersburg/Leningrad, Russia, from 1706 to 1869 CE.

In addition to compiling existing documentary data series, we also rescued a significant amount of data (this includes some series we recently presented in another study^15^). This concerns 137 ice phenology series and 5 precipitation series (Fig. 2). Note that some of the rescued data might be available electronically but we did not find it. The single most important source was a compilation of freezing and thawing dates of Russian rivers by Rykachev^16^ (see example in Fig. 3) and a follow-up compilation by Shostakovich^17^. Some (few) series were measured from graphs published in the 1970s where the underlying data were unavailable electronically. Many of the datasets digitized in the 1970s and even 1980s have not made it into the era of electronic publishing and open data policies (a list of rescued series including the sources is given in Supplementary file DOCU-CLIM_Rescued.txt).

### Evaluation

To identify any climate signal contained in documentary climate data, we formulated forward models^18^ based on instrumental global monthly climate fields. These models not only serve for evaluation in this paper but are directly relevant for climate reconstruction approaches including data assimilation. We used temperature fields from BEST^19^ and HadCRUT5^20^, and precipitation fields from GPCC^21^ to extract the time series from the closest grid point to each documentary site. The number of overlapping years between proxy and climate series had to be superior to 20 years (for all African wetness/dryness indices, as an exception, we accepted 10 overlapping years as otherwise no evaluation would have been possible on the entire dataset). The starting date was usually dictated by the start of the reference dataset, the end date by the end of the documentary dataset (see exceptions below), but never later than 1950 CE in order to avoid calibrating a forward model in a period in which climate or environment are no longer comparable with earlier periods.

**Fig. 4:**
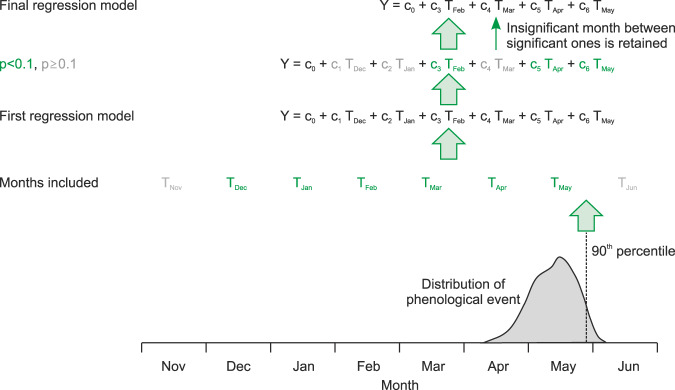
Schematic figure of forward modeling approach.

The forward model took the form of a multiple regression model (see Fig. 4), in which a documentary series was expressed as a linear combination of monthly series of the corresponding driving variable (either temperature or precipitation). If the season or month was specified in the source (e.g., monthly, seasonal, or annual indices), these months were used. If this information was unavailable, we used annual mean values. In the case of events that were indicated as a specific date (e.g., phenological data), we also included lagged predictor variables (i.e., temperature from one or several previous calendar months). The window to be included was determined in a backward selection approach. In this case, the models initially included 6 months prior to the event in question (defined as the 90^th^ percentile), such that an entire growing season could possibly be covered. Then a selection was carried out, retaining only months that were significant at *p* < 0.1. Insignificant months between two significant months were also retained. If no significant months were identified, no model was calculated. For the phenological series covered in Reichen *et al*.^15^, we made use of the more detailed information available. For instance, strongly skewed variables were transformed logarithmically, and we used the reference dataset and reference period given in the paper. The procedure is sketched in Fig. 4.

**Fig. 5:**
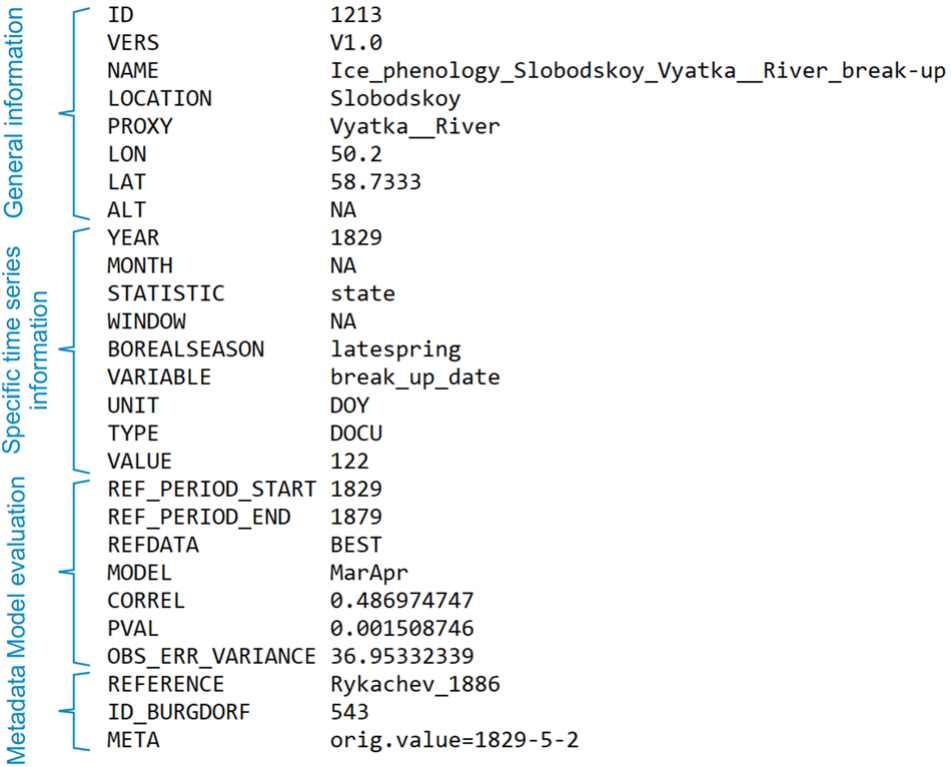
Data format with 27 columns using the example of the first line of series 1213 (transposed for clearer visualization).

We then fitted regression models with a least-squares estimator. As a measure of goodness of fit, we used the correlation between the observed and modelled documentary series, along with the *p*-value. The following information on the evaluation is indicated in the example data file (Fig. 5): the reference period used (1829–1879 CE in this example case), the reference dataset (BEST), the model (monthly mean temperature of March and April; any transformation of variables would be indicated here), correlation, *p*-value, and error variance of the residuals. For some of the ice phenological records, we have also digitized nearby temperature records as the existing global databases did not have any data in close vicinity. These new data have been published in Reichen *et al*.^15^ and Lundstad *et al*.^22^ (10.1594/PANGAEA.940724), and in these cases “REFDATA” is denoted by the label “station”.

It should be noted that, first, the residual error is not a measure of the error of the documentary proxy, but of the difference between the actual observation and the forward modelled observation (regression error). Consequently, it also contains the inherent error in the instrumental climate data and in the interpolation (this is the error required for data assimilation approaches). Second, this evaluation measures the error only in recent times when instrumental climate data are available. As a result, the quality of the documentary data in the earlier period may differ^23^.

For all proxies that do not have a sufficiently long (see thresholds above) overlap with instrumental climate fields or nearby station records, an independent evaluation was not attempted here. This is indicated with an “NA” throughout the evaluation section of the data file. Other methods of evaluation are possible, but this requires more knowledge and hence we refer to the original publications. It would be possible to compare these cases with reconstructions such as EKF400v2^5^, a global, monthly three-dimensional climate reconstruction covering 1600–2003 CE. EKF400v2 is based on an off-line assimilation approach of proxy data (e.g., tree-ring width, maximum late wood density), documentary data, and early instrumental data into an ensemble of atmospheric model simulations. However, in many cases the documentary data were assimilated in EKF400v2 (and hence datasets are not independent), while in cases where no information is locally available, EKF400v2 basically represents a model simulation, so no strong correlation is expected. Accordingly, we use EKF400v2 only in Sect. 4 for a case study.

Some documentary indices continue into the instrumental era as the authors have complemented them with degraded instrumental data or have used instrumental data in addition to documentary data. These data may then not be independent of instrumental data. These values were not removed from DOCU-CLIM. However, in the evaluation conducted in this study, the calibration period in such cases is limited to years before 1900 CE. Where available, information on whether a value was from a documentary source or from degraded observations was added to the “META” column.

## Data Records

**Fig. 6:**
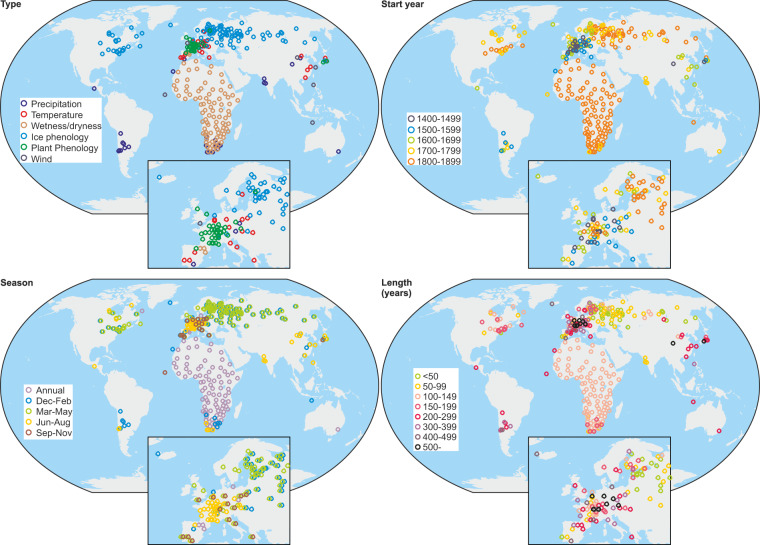
Map of all documentary data categorized according to (top left) the variable, (top right) the start year, (bottom left) the season covered, and (bottom right) the length.

The DOCU-CLIM dataset can be downloaded from the BORIS repository (https://boris-portal.unibe.ch/handle/20.500.12422/207)^24^. The dataset comprises 621 files (note that a monthly index series is split into 12 files), totaling more than 100,000 values (Fig. 6). Information on all series, including links to the original holding, is given in the readme-file of the dataset^24^. The references of the original series are included in this paper (refs. ^16,17,25–129^). The files are in ASCII format with 27 columns and a variable number of lines. The files are structured in a way that allows for straightforward inclusion into data assimilation schemes (see Fig. 5). In each file, one line covers one year. As a consequence, monthly data are stored in 12 files, one for each calendar month. The first seven columns contain information about the series, version number, and geographical location. These are identical for each line in the file. Then come the year, and the month (only given if the record resolution is monthly or seasonal). Where documentary information refers to a specific time (e.g., date of freezing), the month is set to NA. The column “STATISTIC” indicates whether the observation is a state (such as a date of freezing), or a mean value (e.g., a seasonal mean index, in which case the indicated month gives the last month of the averaged interval and the column “WINDOW” the number of months averaged). The column “BOREALSEASON” indicates the closest match to a season: winter (Dec-Feb), spring (Mar-May), summer (Jun-Aug), autumn (Sep-Nov), or annual (for ice freezing and thawing series we additionally used “earlywinter” and “latespring”). The next columns indicate variable name, unit, and type (for all series in this paper, the type is “DOCU”), then follows the column “VALUE” that contains the actual time series values. The next seven columns refer to fitting metrics of the forward model, as described in Sect. 2.2. Finally, the last three columns provide metadata such as a reference, the ID of the corresponding series in Burgdorf^13^, and a column “META” that contains further information (for several entries, they are separated by “|”). In all cases the META column provides the original value (which is often the same as the value itself). Figure 5 provides an example where the freezing date is given in yr-mon-day. For further metadata on the series and collections, the reader is referred to the inventory by Burgdorf^13^.

The file structure is further explained in the supplementary file “DOCU_CLIM_File_Description.txt”. All file names start with “DOCU_CLIM” followed by the ID as six-digit integer, followed by the version number “V1.0” and the name of the variable; all of these elements are separated by an underscore character “_”. As an example, the file shown in Fig. 5 is named: “DOCU_CLIM_001213_V1.0_Ice_phenology_Slobodskoy_Vyatka__River_break-up.txt”. R-code to read the files is given in the Supplementary material.

Most of the document-based climate records are from Europe, which is partly due to our selection criteria. However, there are also records from Asia and North America. Data for Africa mostly concern precipitation^27^. We only have a few documentary records from South America^28,29^ and only two from Australia^30,31^.

One of the advantages of documentary proxy data is that they encompass all seasons (left bottom panel in Fig. 6). For example, plant phenology reflects temperature in spring and summer (sometimes autumn). Ice phenology reflects conditions from late autumn to spring as ice-on dates are associated with autumn and winter air temperatures and ice-off dates are highly correlated to winter and spring air temperatures. Many of the indices such as temperature and precipitation indices for various regions of Europe are seasonal or even monthly. Some documentary data such as wetness/dryness in Africa indicate annual conditions (interpreted as yearly means)^27^.

The earliest records that extend back to the 15^th^ century or further are mainly from Europe, China and Japan (top right panel in Fig. 6). Some records from South America begin as early as the 16^th^ century, and those from North America date back to the 18^th^ century, while the earliest African records start around 1800. Many of the ice phenological data date back to the 1800s, but there are also longer records such as that of Lake Suwa, Japan, beginning in 1443. The oldest records are typically the longest (right panels in Fig. 6), as many continue to the start of instrumental observations or even beyond.

**Fig. 7:**
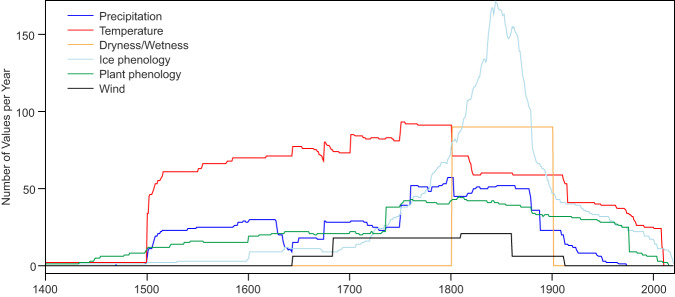
Number of values in the DOCU-CLIM dataset as a function of year and type of proxy.

Many records start after 1500 CE (Fig. 7) and the maximum coverage is in the late 19th century (note that from 1880 onward, no new records were added, although many phenological series start later). Temperature and precipitation indices are the most frequent record type, and wind indices^31–34^ the least. However, the numbers for different types vary in time (Fig. 7). During the 19^th^ century, when many weather stations were already measuring temperature in Europe, the number of documentary temperature series decreases and ice phenological records dominate.

**Fig. 8:**
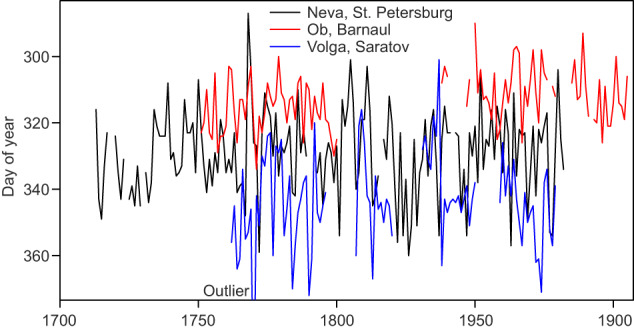
Time series of the freeze-up date of three Russian rivers. Note the reverse scale on the y-axis.

As an example of series in DOCU-CLIM, Fig. 8 shows three time series of freeze-up dates of Russian rivers. Ice phenology is the dominating type of documentary data in DOCU-CLIM during most of the 19^th^ century. While the Neva series is continuous, the one from the Ob in Barnaul has a long gap. The series from the Volga in Saratov are shorter and contain an outlier (freeze-up date: 14 Feb). Note that outliers were not filtered out for the following evaluation.

## Technical Validation

For many of the time series, a technical validation was performed by the original authors. These evaluations include arguably the most specific, expert judgement, combining local knowledge on both the historical sources and the local-to-regional climate characteristics. Readers are encouraged to consult the original publication for specific details (references are indicated in the inventory file as well as in the data files; a link is also given to the inventory by Burgdorf^13^ where further information is available).

**Fig. 9:**
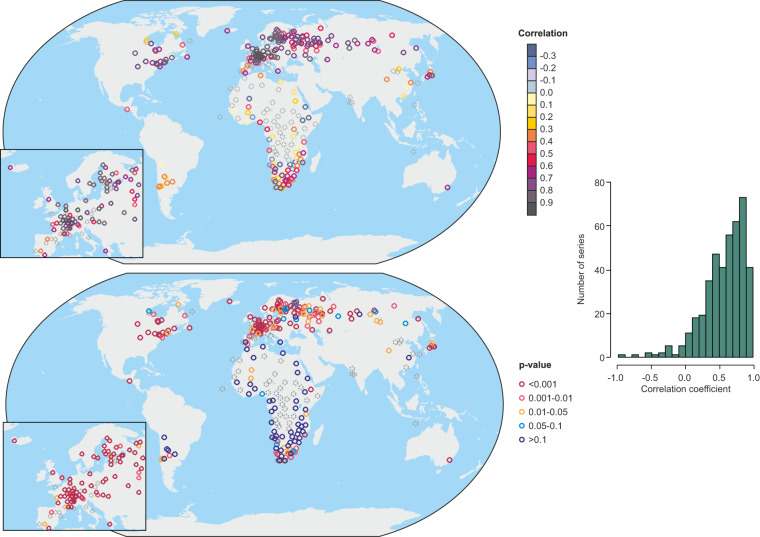
Map of Pearson correlation coefficients between documentary data and forward modelled data (top), *p*-values (bottom) and histogram showing the distribution of the correlation coefficients. Grey dotted circles indicate series where no evaluation was possible.

Here we report the results of our independent validation, as described in Section 2. In Fig. 9 we show correlations for forward models that we calibrated in gridded instrument-based datasets. All records that have no overlap with observations (and thus evaluation was not possible) are denoted by grey dotted circles.

We found robust and highly significant correlations across Europe, North America and Asia. Many of them are related to derived indices, plant phenology, or ice phenology. Somewhat lower but still highly significant correlations are found over South America. Spatially-varying correlations are found over Africa, where most of the series were evaluated based on only 10 years of overlap. Moreover, the precipitation dataset^21^, which the documentary data were compared with, may have substantial errors in these pre-1900 years. However, several significant correlations can be found in locations including southern Africa and Australia.

The distribution of the correlations (Fig. 9) shows that an overwhelming number of series exhibits correlations above 0.5 and the peak of the distribution occurs at correlations between 0.7 and 0.9, which is higher than the correlations found for forward modelling of tree rings^7^. The highest correlations are observed for temperature indices, which are however often not fully independent from instrumental data in the overlapping period. Very high correlations are however, also found for ice phenological data and grape harvest dates.

The evaluation of the 421 records with models demonstrates that many documentary series have significant potential for quantitative applications. However, the series that were not evaluated (due to the absence of instrumental series in the vicinity, or total lack of overlap) will require further investigation and consultation in the literature before incorporation into a climate reconstruction.

## Usage Notes

The DOCU-CLIM dataset^24^ provides climate information from documentary data with the main aim of facilitating climate reconstructions. The dataset contains information on the correlation with corresponding forward models. This information should be carefully considered before using the data. Although climate reconstruction is the primary aim, the data can also be used in the form of individual time series.

**Fig. 10:**
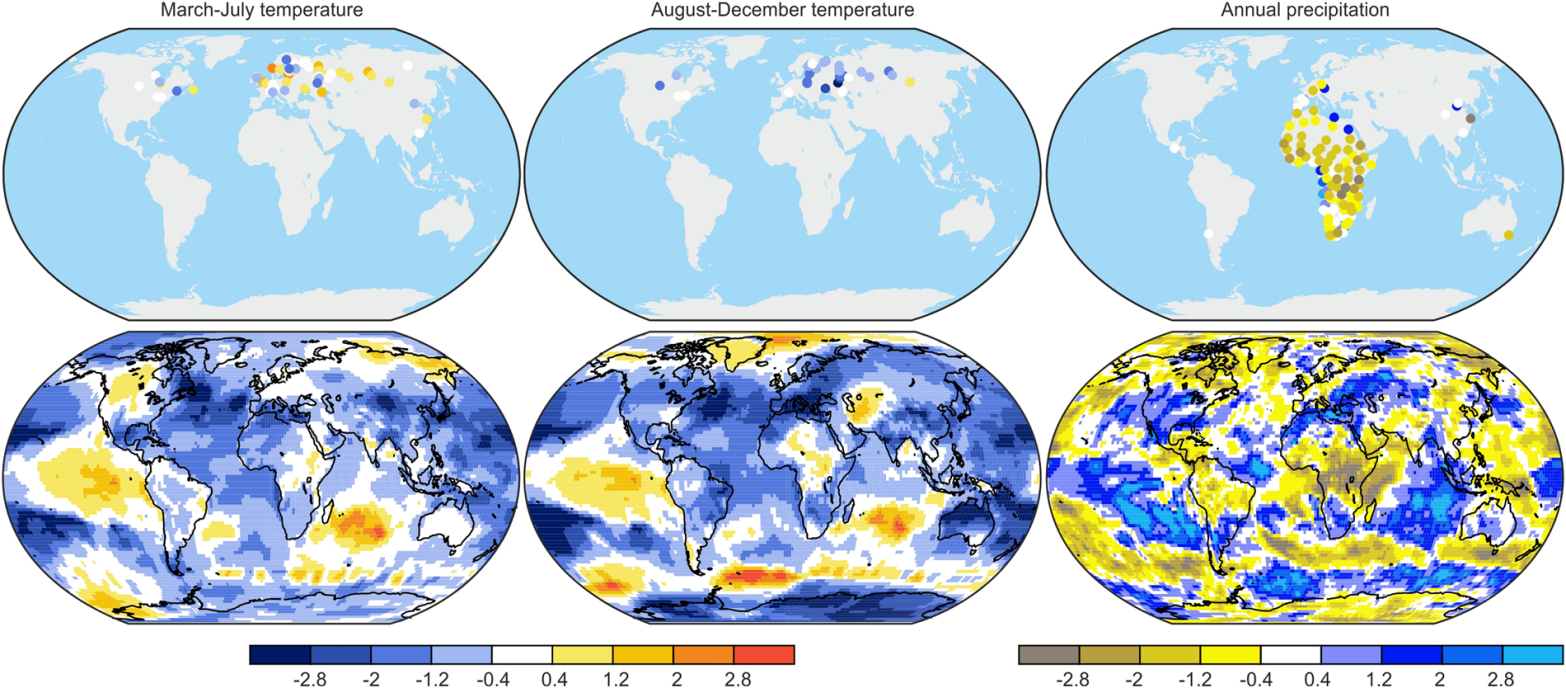
Standardized climatic anomalies in 1835 (with respect to the period 1841–1870) for (top) documentary data and (bottom) EKF400v2. Variables include temperature in (left) spring to summer and (middle) autumn to early winter and (right) annual precipitation.

To demonstrate the potential of this new documentary dataset for quantitative climate analysis, we present a case study for the year 1835. In January 1835, the volcano Cosigüina in Nicaragua erupted and released massive amounts of sulfuric aerosols into the atmosphere. It is considered one of the largest historical volcanic eruptions in the Americas and led to widespread environmental impacts^130^. We investigated temperature-related series, and precipitation or wetness/dryness-related time series, for the year 1835 CE. For temperature, we differentiated two extended seasons: boreal spring to summer (March to July), and autumn to early winter (August to December), as this division fits best with the material contained in the documentary data (thawing dates and spring/summer phenology, freezing dates and autumn phenology). Monthly series were averaged to these seasons. For precipitation and wetness/dryness indices, we considered annual indices or means of all seasons. As a reference we chose the period 1841–1870 CE (retaining only series with more than 20 years of data in this period) and standardized the series with respect to this reference. Finally, the sign of series was adjusted such that, for temperature, positive indicates warmer conditions (e.g., spring flowering or thawing dates were multiplied by −1 as earlier dates indicate warming; the sign of freezing dates was kept as early freezing indicates low temperatures). Series that were already assimilated in EKF400v2 were excluded from this analysis. We then compared these anomalies to the EKF400v2 reanalysis where we performed the same procedure with global monthly fields. Seasonal and annual averages were calculated, and the fields for 1835 were presented as standardized anomalies from the 1841–1870 base period. Figure 10 shows the standardized anomalies of the documentary proxies (top row) and EKF400v2 (bottom row) for the year 1835 CE. The two sets of data are entirely independent, and they are plotted on the same scale.

EKF400v2 shows a general cooling that is arguably related to the volcanic eruption. However, not all regions cooled in all seasons. In boreal spring and summer, Europe and northern Eurasia have standardized anomalies around zero, and in some regions, temperature anomalies are positive. Although EKF400v2 assimilates no ice phenology from Siberian rivers except for the Angara in Irkutsk, these regions also showed neutral or slightly warm conditions. During autumn and early winter, the documentary data (particularly the early freezing of rivers) suggest a general cooling across the northern mid-latitudes. This coldness corresponds well with the EKF400v2 anomaly fields. Finally, precipitation in EKF400v2 indicates drying in most parts of Africa and wetting around the Mediterranean. This pattern is also observed in most of the documentary data in Africa (none of which were assimilated into EKF400v2). The large-scale cooling and the drying of areas influenced by the African monsoon agree with the expected effects of a tropical volcanic eruption^131^. Overall, our analysis shows that our documentary dataset (DOCU-CLIM)^24^ can capture spatial climate variability associated with the prominent volcanic eruption of 1835.

The DOCU-CLIM dataset^24^ can be used for climate reconstruction, particularly for data assimilation, which can make full use of the data and the metadata provided on the forward modeling. Some documentary time series could not be validated and should be further analyzed. DOCU-CLIM is a global dataset^24^ and can now be combined with other multi-proxy compilations such as the PAGES 2k^6^ datasets, or instrumental datasets such as H-CLIM^22^, to generate new climate reconstructions.

Care should be taken when evaluating the series for trends. We have not analyzed the suitability of the records for trend analyses and advise testing this further before using the dataset for this purpose. The answer may well depend on the proxy type considered (phenological data, thermal index, etc.). Possible future updates of the DOCU-CLIM dataset may offer the data in a range of other existing data formats^132^.

## Data Availability

R code for generating the plots in this paper, for reading in all files and extracting desired information, and for the forward modeling is available from https://github.com/sbroennimann/DOCU-CLIM.
